# Coendangered hard-ticks: threatened or threatening?

**DOI:** 10.1186/1756-3305-4-71

**Published:** 2011-05-09

**Authors:** Andrei Daniel Mihalca, Călin Mircea Gherman, Vasile Cozma

**Affiliations:** 1 Department of Parasitology and Parasitic Diseases, University of Agricultural Sciences and Veterinary Medicine Cluj-Napoca, Calea Mănăştur 3-5, Cluj-Napoca, 400372, Romania

## Abstract

The overwhelming majority of animal conservation projects are focused on vertebrates, despite most of the species on Earth being invertebrates. Estimates state that about half of all named species of invertebrates are parasitic in at least one stage of their development. The dilemma of viewing parasites as biodiversity or pest has been discussed by several authors. However, ticks were omitted. The latest taxonomic synopses of non-fossil Ixodidae consider valid 700 species. Though, how many of them are still extant is almost impossible to tell, as many of them are known only from type specimens in museums and were never collected since their original description. Moreover, many hosts are endangered and as part of conservation efforts of threatened vertebrates, a common practice is the removal of, and treatment for external parasites, with devastating impact on tick populations. There are several known cases when the host became extinct with subsequent coextinction of their ectoparasites. For our synoptic approach we have used the IUCN status of the host in order to evaluate the status of specifically associated hard-ticks. As a result, we propose a number of 63 coendangered and one extinct hard-tick species. On the other side of the coin, the most important issue regarding tick-host associations is vectorial transmission of microbial pathogens (i.e. viruses, bacteria, protozoans). Tick-borne diseases of threatened vertebrates are sometimes fatal to their hosts. Mortality associated with pathogens acquired from ticks has been documented in several cases, mostly after translocations. Are ticks a real threat to their coendangered host and should they be eliminated? Up to date, there are no reliable proofs that ticks listed by us as coendangered are competent vectors for pathogens of endangered animals.

## Biodiversity or pest?

In their review on tick-host specificity from 1982, Hoogstraal & Aeschlimann wrote: "*As biomedical researchers, we are charged with the task of improving the quality of human life and welfare precisely, reducing risks of disease, irritation, and debilitation resulting from parasitism by ticks*" [1]. From strict medical point of view they might be questionably right. But, ticks, as all parasitic species, are part of global biodiversity which according to current trends should be preserved.

The overwhelming majority of conservation projects in the animal kingdom are focused on vertebrates, despite most of the species on Earth being invertebrates. Estimates state that about half of all named species of invertebrates are parasitic in at least one stage of their development [2]. The dilemma of viewing parasites as biodiversity or pest has been discussed by several authors, regardless if it is about animal parasites [3-8]. General human perception of parasites is usually negative and several dictionaries derogatorily associate this concept with exploitation. Among all parasites, ticks, along with other ectoparasites seem to have one of the most negative reputations [9].

## Extant or extinct?

Ticks (suborder Ixodida) are obligate blood-sucking acarines attacking a wide variety of hosts from all tetrapod vertebrate classes (Amphibia, Reptilia, Aves and Mammalia). Three families are currently recognized: Ixodidae (hard ticks), Argasidae (soft ticks) and Nuttalliellidae. The latest taxonomical synopses of the group [10-12] updated by [13] consider valid 700 non-fossil species in Ixodidae (for a review of fossil ticks see [14]). Though, how many of the 'non-fossil' species are still extant is almost impossible to tell, as many of them are known only from type specimens in museums and were never collected since their original description.

Generally, extinction is considered to have four main causes: habitat loss, species invasion, overkill and cascades of extinctions [15]. Cascades of extinctions (or coextinctions) are in most situations cases of habitat loss in species for which the habitat is another species, like the case of mutualists, commensals and parasites. In the case of most symbiotic interactions the extinction of the host could result in the extinction of several associated species [16]. Ticks are no exception.

## Narrow host specificity makes ticks co-endangered

After the concept of '*coextinction *was intuited by Darwin in 1862 and introduced in scientific literature in 1993 [17], the term '*coendangered*' arose logically within the next years [18], when estimates stated that 6300 symbiotic species are coendangered with their associated organisms. Nevertheless, the review omitted several groups of parasites like protozoans, cestodes, trematodes, most nematodes, acanthocephalans, fleas, ticks, whale lice etc. Therefore, the number of coendangered parasites could be much higher. For ectoparasites, including ticks, not only the endangered status of the host makes them endangered. As part of conservation efforts of threatened vertebrates, actions often involve artificial breeding, re-introduction or relocations. During these processes, a common practice is the removal of external parasites, with devastating impact on their population [19]. Several cases are documented. One relevant example is of the louse *Colpocephalum californici *(now extinct) which were intentionally removed from the endangered California condor, *Gymnogyps californianus *during the captive breeding project at Los Angeles Zoo [20].

In the case of parasites, the coendangered status applies with predilection to species with high host-specificity. Ticks are distributed worldwide from the Arctic to tropical regions. Their geographical distribution is related to the range of their host(s) with the highest diversity in tropical regions. Host specificity in ticks is still a debated issue. In some tick species, the host specificity was evaluated by more or less complex experimental trials, but in the majority of the situations this label comes solely from field reports on tick-host associations. In the first situation, one of the most studied species is the cattle tick, *Rhipicephalus *(*Boophilus) microplus*. Several hypotheses were incriminated to explain host specificity in ticks: adaptation by the tick to the particular properties of host's skin, specific sensory stimulus to attachment, specific ability of the tick to evade the host's immune responses or dietary specificity [21-23]. Based on a review of experimental evidence or ecological observations, about 85% of the tick species are considered to have a certain degree of host specificity, especially in their adult stage [1]. However, sometimes ecological specificity (habitat dependence) could explain the apparent specific host association in ticks, reducing the access of certain tick species to a limited number of vertebrate species [24].

The first and single review so far on tick conservation [19] proposed 42 species of Ixodidae as candidates for the endangered status. Following this idea, the echidna tick *Bothriocroton oudemansi *was listed as coendangered with its host [25]. Similar opinions are available for other groups of parasites. The conservation status of myiasis causing Oestrid flies was discussed recently in detail [6]. In this review, the authors grouped the endangered parasitic flies into three categories, by the cause of possible extinction: treatment-induced, coextinction and neglected, listing a total number of 39 bot-flies. A synoptic review on coextinct lice of birds and mammals is also available [26].

## A synopsis of ticks proposed for coendangered status

The International Union for Conservation of Nature (IUCN) classifies organisms into seven categories, according to their conservation status [27]. Additionally, some species have entries in the red list database, but their status is listed as data deficient. Furthermore, many species are not present at all in the IUCN database, meaning they have not been evaluated to date. For our synoptic approach we have used the IUCN status of the host in order to evaluate the status of specifically associated tick parasites, following the algorithm in Table 1. The list of valid ticks species used was according to the latest taxonomical reviews of the group [10,11,13].

**Table 1:** Algorithm used for proposal of tick conservation status

Proposed status of the tick	IUCN status of the host
Extinct	EX, EW

Coendangered	CR, EN, VU

EX - Extinct; EW - Extinct in the Wild; CR - Critically Endangered; EN - Endangered; VU - Vulnerable

Extinction of single host species could result in the immediate extinction of several associated species (parasites, commensals, mutualists) [16]. In the case of Ixodidae, there are certain threatened vertebrates which host more than one tick species. For instance, the extinction of the sambar deer (*Rusa unicolor*) could lead to the coextinction of four specifically associated ticks. Moreover, ticks harbor themselves internal symbiotic microorganisms, most of them not studied. Thus, the resulted chain of extinctions is much more complex and difficult to estimate.

Our synoptic evaluation of ticks specifically associated with their threatened host revealed a number of 63 coendangered species (Tables 2 and 3).

**Table 2:** Summary of Ixodidae (hard ticks) proposed to be considered coendangered

Genus	Number of valid species	Number of coendangered species	Host cathegory		
			
			Reptiles	Birds	Mammals
*Amblyomma*	130	31	19	1	11

*Anomalohimalaya*	3	0	-	-	-

*Bothriocroton*	7	1	-	-	1

*Cosmiomma*	1	1	-	-	1

*Dermacentor*	34	3	-	-	3

*Haemaphysalis*	166	9	-	-	9

*Hyalomma*	27	2	1	-	1

*Ixodes*	243	16^a^	-	4	12

*Margaropus*	3	0	-	-	-

*Nosomma*	2	0	-	-	-

*Rhipicentor*	2	0	-	-	-

*Rhipicephalus*	82	0	-	-	-

TOTAL	700	63	20	5	38

a - *Ixodes nitens *which we list as extinct is not included

**Table 3:** Host associations of Ixodidae proposed to be coendangered

Species	Distribution	Main hosts	IUCN status of host
*Ixodes anatis *Chilton, 1904	New Zeeland	*Apteryx mantelli*	EN
		
		*Apteryx australis*	VU

*I. dendrolagi *Wilson, 1967	New Guinea	*Dendrolagus matschiei*	EN
		
		*Dendrolagus dorianus*	VU

*I. diomedeae *Arthur, 1958	Tristan da Cunha Islands	*Thalassarche chlororhynchos*	EN

*I. galapagoensis *Clifford and Hoogstraal, 1980	Galapagos	*Aegialomys galapagoensis*	VU

*I. lemuris *Arthur, 1958	Madagascar	*Eulemur macaco*	VU

*I. montoyanus *Cooley, 1944K	South America	*Pudu puda*	VU

*I. moscharius *Teng, 1982	Tibet	*Moschus berezovskii*	EN

*I. moschiferi *Nemenz, 1968	Nepal, China	*Moschus berezovskii*	EN

*I. murreleti *Cooley and Kohls, 1945	Coronados Islands	*Synthliboramphus hypoleucus*	VU

*I. percavatus *Neumann, 1906	Tristan da Cunha Islands	*Thalassarche chlororhynchos*	EN

*I. schillingsi *Neumann, 1901	Africa	*Colobus polykomos*	VU

*I. stilesi *Neumann, 1911	Chile	*Pudu puda*	VU

*I. taglei *Kohls, 1969	Chile	*Pudu puda*	VU

*I. tapirus *Kohls, 1957	Central and South America	*Tapirus pinchaque*	EN
		
		*Tapirus bairdii*	EN

*I. vestitus *Neumann, 1908	Australia	*Myrmecobius fasciatus*	EN

*I. zaglossi *Kohls, 1960	New Guinea	*Zaglossus bruijni*	CR

*Haemaphysalis borneata *Hoogstraal, 1971	Malaysia	*Rusa unicolor*	VU

*H. capricornis *Hoogstraal, 1966	Thailand	*Capricornis sumatraensis*	VU

*H. goral *Hoogstraal, 1970	China	*Nemorhaedus griseus*	VU

*H. kopetdaghica *Kerbabaev, 1962	Asia	*Capra aegagrus*	VU

*H. moschisuga *Teng, 1980	China	*Moschus berezovskii*	EN

*H. pentalagi *Pospelova-Shtrom, 1935	Japan	*Pentalagus furnessi*	EN

*H. psalistos *Hoogstraal, Kohls and Parrish, 1967	Philippines	*Rusa unicolor*	VU

*H. sambar *Hoogstraal, 1971	India	*Rusa unicolor*	VU

*H. vietnamensis *Hoogstraal and Wilson, 1966	Asia	*Rusa unicolor*	VU

*Dermacentor circumguttatus *Neumann, 1897	Africa	*Loxodonta africana*	VU

*D. latus *Cooley, 1937	Central America	*Tapirus bairdii*	EN

*D. rhinocerinus *(Denny, 1843)	Africa	*Diceros bicornis*	CR
		
		*Ceratotherium simum*	NT

*Hyalomma aegyptium *(Linnaeus, 1758)	Africa, Eurasia	*Testudo graeca*	VU
		
		*Testudo horsfieldi*	VU

*H. rhipicephaloides *Neumann, 1901	Middle East	*Gazella gazella*	VU

*Bothriocroton oedemansi *(Neumann, 1910)	New Guinea	*Zaglossus bruijni*	CR

*Cosmiomma hippopotamensis *(Denny, 1843)	Africa	*Hippopotamus amphibius*	VU *0*.
		
		*Diceros bicornis*	CR

*Amblyomma albopictum *Neumann, 1899	West Indies	*Cyclura lewisi*	CR
		
		*Cyclura cornuta*	VU

*A. antillorum *Kohls, 1969	West Indies	*Cyclura pinguis*	CR
		
		*Iguana delicatissima*	VU
		
		*Cyclura carinata*	EN

*A. argentinae *Neumann, 1905	Argentina	*Chelonoidis chilensis*	VU

*A. boeroi *Nava et al., 2009	Argentina	*Catagonus wagneri*	EN

*A. chabaudi *Rageau, 1964	Madagascar	*Pyxis arachnoides*	EN
		
		*Astrochelys radiata*	EN

*A. clypeolatum *Neumann, 1899	Asia	*Geochelone platynota*	EN
		
		*Indotestudo elongata*	EN

*A. coelebs *Neumann, 1899	Central and South America	*Tapirus bairdii*	EN
		
		*Tapirus terrestris*	VU

*A. crassum *Robinson, 1926	South America	*Chelonoidis denticulata*	VU

*A. crenatum *Neumann, 1899	Java	*Rhinoceros sondaicus*	CR

*A. cruciferum *Neumann, 1901	West Indies	*Cyclura cornuta*	VU

*A. darwini *Hirst and Hirst, 1910	Galapagos	*Amblyrhynchus cristatus*	VU

*A. geochelone *Durden, Keirans and Smith, 2002	Madagascar	*Astrochelys yniphora*	CR

*A. humerale *Koch, 1844	South America	*Chelonoidis denticulata*	VU

*A. incisum *Neumann, 1906	Central and North America	*Tapirus terrestris*	VU

*A. javanense *(Supino, 1897)	Asia	*Manis javanica*	EN
		
		*Manis pentadactyla*	EN

*A. komodoense *(Oudemans, 1929)	Indonesia	*Varanus komodoensis*	VU

*A. latepunctatum *Tonelli-Rondelli, 1939	South America	*Tapirus terrestris*	VU

*A. macfarlandi *Keirans, Hoogstraal and Clifford, 1973	Galapagos	*Chelonoidis nigra*	VU

*A. multipunctum *Neumann, 1899	South America	*Tapirus *sp.	EN/VU^1^

*A. papuanum *Hirst, 1914	Australia	*Casuarius casuarius*	VU

*A. personatum *Neumann, 1901	Africa	*Diceros bicornis*	CR

*A. pilosum *Neumann, 1899	Galapagos	*Chelonoidis nigra*	VU

*A. postoculatum *Neumann, 1899	Australia	*Lagostrophus fasciatus*	EN

*A. rhinocerotis *(de Geer, 1778)	Africa	*Diceros bicornis*	CR
		
		*Ceratotherium simum*	NT

*A. robinsoni *Warburton, 1927	Indonesia	*Varanus komodoensis*	VU

*A. supinoi *Neumann, 1905	Asia	*Indotestudo elongata*	EN
		
		*Heosemys spinosa*	EN
		
		*Heosemys depressa*	CR

*A. tholloni *Neumann, 1899	Africa	*Loxodonta africana*	VU

*A. torrei *Perez Vigueras, 1934	West Indies	*Cyclura lewisi*	CR

*A. tuberculatum *Marx, 1894	USA	*Gopherus polyphemus*	VU

*A. usingeri *Keirans, Hoogstraal and Clifford, 1973	Galapagos	*Chelonoidis nigra*	VU

*A. williamsi *Banks, 1924	Galapagos	*Conolophus subcristatus*	VU

1 - The host for *A. multipunctum *was listed only as *Tapirus *sp. Only four species of genus *Tapirus *are known, three of which are endangered and one vulnerable.

CR - Critically Endangered; EN - Endangered; VU - Vulnerable; NT - Near Threatened

Most species included in our review (n = 31) belong to genus *Amblyomma *Koch, 1844. Their host specificity is high, especially in their adult stage [1] which makes them candidates for extinction if their hosts become extinct. Within the genus *Ixodes*, we propose 16 species, parasitic on tropical birds or mammals, as coendangered. All coendangered species (n = 9) from the genus *Haemaphysalis *Koch, 1844 are restricted to Asian threatened mammals. Only three species of the genus *Dermacentor *Koch, 1844 are included in our synopsis. The genus *Hyalomma *Koch, 1844, parasitic on mammals and tortoises includes two coendangered species. The genus *Bothriocroton *Keirans, King and Sharrad, 1994, recently erected to genus level, was initially described as a subgenus of the former genus *Aponomma *(now synonym of *Amblyomma*) [12]. Seven species are currently included here, all with Australian distribution, with a single species coendangered (*B. oedemansi*). The monospecific genus *Cosmiomma *Schulze, 1919 is found on large threatened mammals from Africa, hence its single species, *Cosmiomma hippopotamensis *is considered coendangered.

### Coendangered ticks of reptiles

Twenty species of coendangered ticks are proposed from those specifically associated with reptiles (Tables 2 and 3). Threatened chelonians harbor 12 of them (11 in the genus *Amblyomma *and 1 in the genus *Hyalomma*). Ten of these chelonian ticks are specifically associated with terrestrial species of the Testudinidae family. On the other hand, *Amblyomma supinoi*, which seems to have less host specificity, has all reported hosts being threatened chelonians (Testudinidae, Geoemydidae) from Asia. The only coendangered tick species of chelonians from Eurasia and Northern Africa is *Hyalomma aegyptium*, parasitic on tortoises of the genus *Testudo*. We can group the eight coendangered ticks of lizards into two major groups (all in the genus *Amblyomma*), based on the taxonomic and biogeographic data of their host: (i) ticks of Iguanidae endemic to West Indies and Galapagos and (ii) ticks of Varanidae from Indonesia.

### Coendangered ticks of birds

Birds harbor five species which we list as coendangered. Four of them belong to the genus *Ixodes *and are non-questing nest ticks parasitic on endangered or vulnerable birds; they were reported exclusively from island habitats (Tables 2 and 3). The Atlantic yellow-nosed albatross (*Thalassarche chlororhynchos*), which nests solely on a few islands from the Atlantic Ocean. is the only recorded host for two species of coendangered ticks. Two species of threatened kiwi birds (genus *Apteryx*) are the only known hosts of *Ixodes anatis *in New Zeeland. The fourth bird-associated *Ixodes *listed here as coendangered is *Ixodes murreleti *found specifically on the Xantus's murrelet (*Synthliboramphus hypoleucus*) in the Coronados Islands. The principal host of *Amblyomma papuanum *is the vulnerable flightless Southern cassowary (*Casuarius casuarius*) from Papua New Guinea.

### Coendangered ticks of mammals

The 38 species of coendangered ticks associated with mammals belong to several genera (Table 2): *Ixodes, Haemaphysalis, Dermacentor, Hyalomma, Bothriocroton, Cosmiomma *and *Amblyomma*.

Two species are parasitic on an egg-laying mammal, the critically endangered Western long-beaked echidna (*Zaglossus bruijnii*) in New Guinea. The other three species are found on threatened marsupials from Australia or New Guinea. South and Central American tapirs (genus *Tapirus*) are hosts to six coendangered ticks in the genera *Ixodes, Dermacentor *and *Amblyomma*. Seven species of ticks are specific parasites of elephants, rhinoceros and hippopotamus. Although the distribution range of these hosts is still wide, antiparasitic treatments during translocations pose a large problem to the survival of associated ticks [19]. Several threatened South American and Asian even-toed ungulates (order Artiodactyla) are specific hosts to 15 species of coendangered ticks. Five additional coendangered tick species are each parasitic on species from five other mammalian orders. *Ixodes galapagoensis *on a rodent in Galapagos, *Ixodes lemuris *on a lemur in Madagascar, *Ixodes schillingsi *on a primate in Africa, *Haemaphysalis pentalagi *on a lagomorph in Japan and *Amblyomma javanense *on pangolins in Asia.

## Extinct ticks

Ticks described from fossil deposits are omitted. We consider extinct one species, namely *Ixodes nitens*, described from two female ticks collected on *Rattus macleari *on Christmas Island. The last report of the host species was in 1903 [28]. As the other endemic rat species *Rattus nativitatis *(sympatric with *R. macleari*), did not harbor *I. nitens*, we can assume this tick was specifically associated with its type host. Thus, we exclude the possibility that *I. nitens *might have re-adapted as a parasite of the introduced black rats, *Rattus rattus *[29]. However, no direct evidence is available.

## Are endangered hosts endangered because of ticks?

Probably the most important issue regarding tick-host associations is vectorial transmission of microbial pathogens. Ticks are able to transmit viruses, bacteria and protozoans to a variety of hosts. One of the most pathogenic tick-borne microbes are piroplasms (genera *Babesia *and *Theileria*). The potential impact of babesiosis on conservation actions was discussed mainly as a consequence of stress-mediated relapse of chronic infections during translocation [30]. Otherwise, tick-borne diseases of threatened vertebrates are rarely fatal to their hosts. The following accounts consider only reports from hosts of coendangered ticks. Mortality associated with pathogens (*Babesia bicornis *and *Theileria bicornis*) acquired from ticks has been documented in black rhinoceros in Tanzania and South Africa [31-34]. The specific vector for these two haemoprotozoans is not known, but *Dermacentor rhinocerinus *and *Amblyomma rhinocerotis *were suggested [34]. Hence, extinction of these ticks is expected to result in the eradication of disease caused by *B. bicornis *and *T. bicornis*. A recent study from South Africa showed that 36.41% white rhinoceros were infected with *Theileria bicornis *and 9.23% with *Theileria equi *[35]. However, no pathology associated with the infection was recorded in white rhinoceros. *Babesia loxodontis *was described from asymptomatic African elephants, *Loxodonta africana *[36]; babesiosis in Asian elephants can be associated with weakness, fever, jaundice, constipation and haemoglobinuria [37]. *Babesia pattoni *was reported in *Rusa unicolor *but no associated pathology was described [38].

So far, no tick-borne diseases with impact on the health of threatened birds or reptiles have been described. Asymptomatic infections with *Hemolivia mauritanica *have been reported in *Testudo graeca *over its distribution range [39]. The zoonotic bacterial pathogen *Anaplasma phagocytophilum *have been isolated in ticks *Amblyomma flavomaculatum *collected on monitor lizards (*Varanus exanthematicus*) [40]. Although neither the host nor the ticks are endangered, there is high probability that other *Amblyomma *species could transmit *Anaplasma *to lizards of genus *Varanus*.

## Conclusions

Expectedly or not, we came back to the question of Hoogstraal and Aeschlimann from the beginning of this paper. Should we decide on conservation of rare ticks? Or are they a real threat to their coendangered host and should be eliminated? Ticks as such are not dangerous. Disease, if present, is in most of the situations caused by vectored microbes. Moreover, pathology induced by tick-borne diseases in wild animals is seldom dangerous and is usually related to supplemental stressing factors (i.e. translocation). Last but not least, there is no proof to date that ticks listed by us as coendangered are competent vectors for pathogens of endangered animals.

Nevertheless, IUCN should reconsider the criteria of indexing species in its database as threatened. All symbiotic species (mutuals, commensals, parasites) specifically associated with their host should be listed as co-endangered. As previously suggested [3], some host-specific parasites are more endangered than their host. Moreover, parasites have their own evolutionary importance, and as suggested even in the early 1990's, parasites should have equal rights with their host [3,5].

## Competing interests

The authors declare that they have no competing interests.

## Authors' contributions

ADM - the idea of the manuscript, the intellectual content and wrote the text, reference on reptiles; CMG - reference research on part of the mammals and birds; VC - reference research on mammals and contributed to the text writing, mainly the piroplasm related paragraphs. All authors read and approved the final manuscript.
